# An extended miRNA repertoire in *Rattus norvegicus*

**DOI:** 10.3389/fbinf.2025.1545680

**Published:** 2025-03-10

**Authors:** Julienne Lehmann, Ali Yazbeck, Jörg Hackermüller, Sebastian Canzler

**Affiliations:** ^1^ Department Computational Biology and Chemistry, Helmholtz Centre for Environmental Research - UFZ, Leipzig, Germany; ^2^ Department of Internal Medicine and Pediatrics, HIV Cure Research Center, Ghent University Hospital, Ghent University, Ghent, Belgium; ^3^ Medical Faculty, Institute of Pathology, University of Cologne, Cologne, Germany; ^4^ Department of Computer Science, Leipzig University, Leipzig, Germany

**Keywords:** miRNA, micro RNA, ncRNA, non-coding RNA, homology search, *Rattus norvegicus*

## 1 Introduction

MicroRNAs (miRNAs) are small, non-coding RNA molecules, approximately 22 nucleotides in length, that play crucial roles in the regulation of gene expression. They function primarily by binding to complementary sequences in the 3′ untranslated regions (UTRs) of target messenger RNAs (mRNAs), leading to mRNA degradation or translational repression (Bartel, 2004; Bushati and Cohen, 2007). Through this mechanism, miRNAs are involved in various biological processes, including development, differentiation, proliferation, and apoptosis (Ambros, 2004). The importance of miRNAs as regulatory elements is furthermore emphasized by their involvement in various diseases, particularly cancer, where they can act as either oncogenes or tumor suppressors (Budakoti et al., 2021; Hussen et al., 2021).

MicroRNAs are transcribed as primary miRNAs (pri-miRNAs) and processed into precursor miRNAs (pre-miRNAs), which are typically around 70 nucleotides long and form hairpin structures (Bartel, 2004). The miRNA duplex is generated from this precursor, consisting of a guide strand (mature miRNA) and a passenger strand (mature*). The mature miRNA is incorporated into the RNA-induced silencing complex (RISC) to guide gene silencing, while the mature* strand is usually degraded, although in some cases, it may also be functional (Bartel, 2004; Okamura et al., 2007).

Despite their critical functions, there is a significant discrepancy in the annotation of miRNAs between different model species, notably between rat (*Rattus norvegicus*) and mouse (*Mus musculus*). This discrepancy arises due to differences in sequencing efforts and annotation strategies but also through lineage-specific retroposons playing an essential role in the birth of new miRNA genes (Lehnert et al., 2011). Addressing this gap is essential for leveraging the rat as a model organism in biomedical research, particularly given its widespread use in pharmacology and toxicology studies (Jacob and Kwitek, 2002).

In this study, we corrected several incorrect homology assignments and identified and annotated novel rat miRNAs. Expanding the miRNA repertoire of this crucial model organism will enhance its utility, particularly for toxicological applications, where precise regulatory networks are critical for understanding the molecular basis of toxicity and drug responses.

## 2 Methods

To identify novel rat miRNAs, we utilized MIRfix curated whole precursor miRNA family covariance models as described previously (Yazbeck et al., 2019). We focused on miRNA families that contained at least one mammalian miRNA sequence. The model building was based on miRBase version 21 (Kozomara and Griffiths-Jones, 2014).

We employed infernal v1.1.3 (Nawrocki and Eddy, 2013) to scan the rat genome (Rnor_6.0; Ensembl Release 102, accession number GCA_000001895.4) for potential miRNA candidates using default parameters. We chose Rnor_6.0 as reference since miRBase relies on this assembly for miRNA annotations, ensuring comparability with existing datasets. The candidate miRNAs were then subjected to a series of stringent filtering steps to ensure the accuracy and relevance of the identified sequences: (1) Candidates were filtered based on an e-value cutoff of 0.01 and a bit score threshold of 33, following the recommendations in the infernal tutorial [log_2_ (2 * genome size)]. (2) Duplicated candidates located on unfinished chromosomes were eliminated. (3) Candidates overlapping with repeats annotated by RepeatMasker were excluded (Smit et al., 2015). (4) Candidates that were reverse complements of candidates with smaller e-value were also excluded.

The remaining candidate miRNAs were curated using MIRfix on whole family alignments, which included the newly identified rat candidates. This was followed by an additional manual curation of the alignments involving a check for sequence conservation of mature and/or mature* regions and the assessment of the ability of novel sequences to fold into a hairpin secondary structure.

Potential miRNA candidates were manually assigned names in accordance with their homologous mouse miRNAs. Finally, the novel miRNA sequences were again blasted against the rat genome (Rnor_6.0) to extract the precise genomic coordinates using blastn ^1^. To ensure compatibility with the newer mRatBN7.2 assembly (de Jong et al., 2024) we mapped the coordinates using CrossMap (Zhao et al., 2014).

## 3 Data analysis

For our infernal scan, we utilized 781 mammalian miRNA families (excluding singletons), which included 435 already annotated rat miRNA sequences distributed across 247 miRNA families. This scan resulted in a total of 449 417 significant candidates scattered over 459 miRNA families.

Following a stringent filtering procedure to eliminate duplicates on unfinished chromosomes, overlaps with annotated miRNAs, repeats, and reverse complements, we identified 3521 potential novel miRNAs within 186 families. The three families with the most candidates accounted for nearly 2500 of those potential sequences. These families contain large numbers of annotated mouse sequences (up to 59 in MIPF0000316), hence introducing substantial variability. This circumstance leads to the detection of a high number of candidate sequences. For each of the 186 miRNA families with at least one candidate sequence, we conducted a MIRfix analysis and correction. Additionally, we manually curated the whole family alignments to further refine this set. The final set of new miRNAs in *R. norvegicus* contained 55 novel sequences, that have been uploaded to the European Nucleotide Archive (ENA) at EMBL-EBI with the accession numbers OZ078105–OZ078160^2^. Notably, this included 39 families where no miRNA had previously been annotated in rat.

**FIGURE 1:**
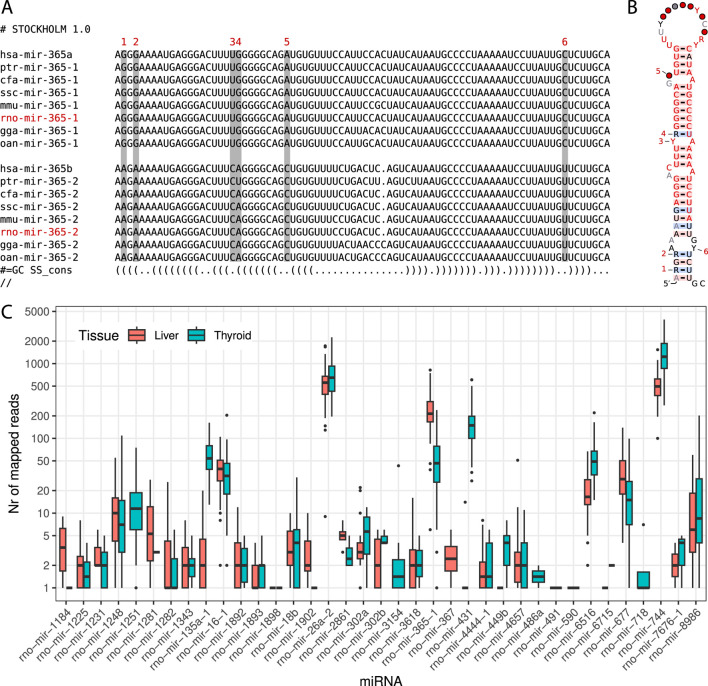
**(A)** MiroRNA sequences of selected model organisms for both subtypes of the miR-365 family. Sequences belonging to subtype ‘a’ or ‘-1’ are shown at the top, while sequences belonging to subtype ‘b’ or ‘-2’ are shown at the bottom. The rat miRNA *rno-mir-365-1* is a new candidate (shown in red). The miRNA *rno-mir-365-2* is already listed in miRBase as *rno-mir-365* and needs to be renamed. Distinct nucleotide differences in the stem region between the two subtypes are indicated above each respective column, numbered from 1 to 6. **(B)** Consensus structure of the miR-365 family containing all 48 sequences of both subtypes. Nucleotide differences are again highlighted with digits from 1 to 6. The secondary structure was visualized using the R2R tool (Weinberg and Breaker, 2011). **(C)** Support for novel miRNA candidates from short RNA-Seq reads. During the XomeTox project, 75 short RNA sequencing libraries were generated from two specific tissues: thyroid and liver (Canzler et al., 2024). Each boxplot summarizes the read counts for individual miRNAs in thyroid and liver tissues on a log scale.

With these discoveries, the updated miRNA repertoire in rats now contains 548 sequences distributed across 341 miRNA families. The complete dataset generated for this study has been deposited at Zenodo^3^ and GitLab^4^ , including sequence files and curated alignments of families with novel miRNA candidates. Additionally, we identified 10 previously annotated rat miRNAs that require renaming due to incorrect homology assignments, as detailed in Table 1. An example of an interesting family requiring the renaming of an existing miRNA and featuring an additional new candidate is illustrated in Figures 1A, B.

**TABLE 1:** Corrected miRNA names and their respective families. Previously annotated miRNAs in miRBase that need to be renamed due to wrong homology assignments.

Previous miRNA name	Updated miRNA name	miRNA family ID
rno-mir-16	rno-mir-16–2	MIPF0000006
rno-mir-365	rno-mir-365–2	MIPF0000061
rno-mir-883	rno-mir-883a	MIPF0000389
rno-mir-17–1	rno-mir-17	MIPF0000001
rno-mir-17–2	rno-mir-106a	MIPF0000001
rno-mir-135a	rno-mir-135a-2	MIPF0000028
rno-mir-199a	rno-mir-199a-2	MIPF0000040
rno-mir-26a	rno-mir-26a-1	MIPF0000043
rno-mir-486	rno-mir-486b	MIPF0000220
rno-mir-3074	rno-mir-3074–1	MIPF0001103

### 3.1 Omics support of new miRNA candidates

Initially, the extended rat miRNA repertoire was generated to provide a more comprehensive miRNA layer for a case study aiming to demonstrate the benefits of multi-omics data integration as part of the CEFIC LRI C5 - XomeTox project^5^. As part of this larger project, we generated short RNA-Seq libraries from 75 rats, examining both thyroid and liver tissues^6^. A detailed description of the methods used is published elsewhere (Canzler et al., 2024).

Using the extended miRNA repertoire, we analyzed the short RNA-Seq data to identify support for these sequences across all distinct samples. We discovered 37 miRNAs with overlapping reads in either or both tissues. Specifically, 35 miRNAs had read support in the thyroid and 32 miRNAs in the liver samples. The read counts for individual miRNA varied significantly, ranging from a few to several thousand per sample, as illustrated in Figure 1C. When miRNAs were detected in both tissues, the read counts were generally comparable.

In summary, this study expands the known miRNA repertoire in *R. norvegicus* by identifying 55 novel miRNAs and correcting misannotated sequences. By bridging the gap between rat and mouse miRNA annotations, this enhanced dataset, which now includes 341 miRNA families, improves the utility of the rat model. These advancements facilitate more comprehensive transcriptomic analyses, particularly in studies where understanding miRNA-regulated pathways is crucial for assessing molecular responses, such as after exposure to toxins and drugs.

## Data Availability

The datasets presented in this study can be found in online repositories. The names of the repository/repositories and accession number(s) can be found in the article/supplementary material.

## Footnotes

1
https://blast.ncbi.nlm.nih.gov/Blast.cgi

2
http://www.ebi.ac.uk/ena/data/view/OZ078105-OZ078160

3
https://doi.org/10.5281/zenodo.12626180

4
https://codebase.helmholtz.cloud/department-computational-biology/xometox/extended_rat_mirna_repertoire

5
https://cefic-lri.org/projects/c5-xometox-evaluating-multi-omics-integration-for-assessing-rodent-thyroid-toxicity/

6
https://www.ncbi.nlm.nih.gov/bioproject/PRJNA695243/
